# The emergence of Exercise Addiction, Body Dysmorphic Disorder, and other image-related psychopathological correlates in fitness settings: A cross sectional study

**DOI:** 10.1371/journal.pone.0213060

**Published:** 2019-04-03

**Authors:** Ornella Corazza, Pierluigi Simonato, Zsolt Demetrovics, Roisin Mooney, Katinka van de Ven, Andres Roman-Urrestarazu, Lili Rácmolnár, Ilaria De Luca, Eduardo Cinosi, Rita Santacroce, Massimo Marini, David Wellsted, Keith Sullivan, Giuseppe Bersani, Giovanni Martinotti

**Affiliations:** 1 Centre for Clinical & Health Research Services, School of Life and Medical Sciences, University of Hertfordshire, Hatfield, United Kingdom; 2 Department of Medico-Surgical Sciences and Biotechnologies, Sapienza University of Rome, Rome, Italy; 3 Dual-Diagnosis Unit, Clinic Parco dei Tigli, Padova, Italy; 4 Department of Clinical Psychology and Addiction, Institute of Psychology, ELTE Eötvös Loránd University, Budapest, Hungary; 5 Social Policy Research Centre, University of New South Wales, Sydney, Australia; 6 Institute of Public Health, University of Cambridge, Cambridge, United Kingdom; 7 Department of International Health, Maastricht University, Maastricht, Netherlands; 8 Hertfordshire Partnership University NHS Foundation Trust, Hatfield, United Kingdom; 9 Department of Neuroscience, Imaging, and Clinical Science, University of Chieti Pescara, Chieti, Italy; 10 Department of Psychiatry, University of Padua, Padua, Italy; Fordham University, UNITED STATES

## Abstract

**Introduction:**

In a society that perpetuates the strive for a perfect appearance, a fit body has become synonymous with success, but simultaneously hard to achieve. This represents a fertile ground for the development of Exercise Addiction (EA) alongside other disorders, such as Body Dysmorphic Disorder (BDD). This study aims to explore the diffusion of EA in fitness settings in the United Kingdom, Italy, Netherlands, Hungary and the previously unexplored association with appearance anxiety, BDD, self-esteem and the use of fitness supplements.

**Methods:**

A large cross-sectional sample (N = 1711) was surveyed in fitness settings using the Exercise Addiction Inventory (EAI), Appearance Anxiety Inventory (AAI) and Rosenberg’s Self Esteem Scale (RSE) in addition to questions surrounding the use of fitness supplements.

**Results:**

Compulsive exercise, appearance anxiety and low self-esteem were present in this sample according to the psychometric measures used (EAI, AAI, RSE). 11.7% scored over the cut off for EA, with alarming peaks in the Netherlands (20.9%) and the United Kingdom (16.1%). 38.5% were found at risk of BDD, mainly female (47.2%). 39.8% used fitness enhancing supplements without medical consultation (95.5%). This cohort of supplement users scored higher in both EAI and AAI. The logistic regression model revealed a strong association between the consumption of sport products and the level of EA across the sample with an odds ratio (OR) of 3.03. Other co-variable factors among female were appearance anxiety (AAI; OR 1.59) and to a lesser extent self-esteem (RSE) (OR 1.08).

**Conclusions:**

This study identified a high risk of EA, appearance anxiety and BDD amongst a cohort of gym users internationally. The previously-unexplored association between these disorders and the unsupervised use of a variety of fitness products, including illicit drugs, highlights the need for informed and integrated responses targeting such vulnerable individuals.

## Introduction

In a fast-paced society that perpetuates the strive for an improved appearance, the ideal of a “perfect” body has been related to success [1]. Such preoccupation with physical appearance has led not only to an objectification of the human body, but also to the development of various appearance-related disorders. This has been confirmed by the growing number of publications on body image perception, body distortion and anxiety disorders over the past three decades [2, 3, 4]. The phenomenon has been linked with the idea of physical exercise as a vehicle to improving body-image as opposed to being motivated by the desire for increased health and well-being. “Train. Eat. Sleep. Repeat”, “Don’t stop until you’re proud”, “The pain you feel today will be the strength you feel tomorrow”, “No pain, no gain” are only few examples of ‘fitspirational’ quotes that are used to inspire a healthy lifestyle through exercise. Millions of texts and selfies posted every day on social networking sites promote a visual representation of ostensibly healthy bodies in a fashionable manner [5, 6, 7, 8, 9, 10, 11, 12]. The most common blueprints for the perfect bodies include being muscular and having a ‘V shape’ for males [13, 14, 15] and having a slender, toned body for females [3, 4]. Although the phenomenon is poorly understood, initial evidence suggests that concerns with physical appearance may represent a continuum from healthy behaviours to psychopathological manifestations related to various forms of anxiety. On this spectrum severe obsessive-compulsive behaviours such as Body Dysmorphic Disorder (BDD) [16, 17] and Muscle Dysmorphia (MD) [18] could be identified. Such disorders could act as precursors or be symptomatic of other clinical conditions, such as eating disorders, mood disorders and addictive behaviours [19, 20, 21, 22].

The construct of Exercise Addiction (EA) is fairly novel and still requires clinical evidence to define the specific characteristics. A recent study by Lichtenstein, Nielsen et al. (2018) shed new light on the relationship between EA, abnormal eating attitudes, anxiety, and depression [23]. However, the concept of EA itself remains controversial with various authors using alternative terms such as "excessive exercising" or "compulsive exercise”. Although EA is not listed in the Diagnostic and Statistical Manual of Mental Disorders (DSM-5) [24], Veale [25] originally classified it as a “primary” disorder characterized by: (1) preoccupation with exercise that has become stereotyped and routine; (2) significant withdrawal symptoms in the absence of exercise (e.g. mood swings, irritability and insomnia); (3) preoccupation that causes clinically significant distress or impairment in physical, social, occupational or other areas of functioning; (4) preoccupation with exercise that is not better accounted for by another mental disorder (e.g. means of losing weight or controlling calorie intake). As a “secondary” disorder, EA may manifest alongside another disorder, resulting in increasingly severe and enduring pathologies [26, 27, 28, 29].

Over the years several theories have tried to explain the conceptual complexity of EA. The first biological based “runner’s high hypothesis” suggested the role of endorphins in producing and sustaining a sensation of strong euphoria after intense exercising [30], while the observation of increased levels of catecholamines post-workout led to the formulation of the “catecholaminergic hypothesis” [31]. Catecholamine levels are involved in the regulation of mood and affect. The later “thermogenic regulation hypothesis” [32] found a point of reconciliation between these physiological and psychological theories. On this regard, it was argued that exercising can act as a “positive reinforcement” because the increased body temperature can reduce anxiety and promote relaxation. The theory was furthered reinforced by the work of Szabo, who stated exercise acted as a coping mechanism; to relieve anxiety and stress (positive reinforcement) or to avoid withdrawal symptoms derived from not exercising (negative reinforcement) [33]. Furthermore, an association between the pathological motivation found amongst exercise addicts and personality traits has been found [34, 35, 36]. Perfectionist and hard-driven individuals with obsessive-compulsive traits are more likely to develop habits relating to EA [37, 38, 39].

Some authors emphasised the connection between EA and body-image concerns such as BDD [1, 15], which has recently been described in the “obsessive-compulsive” chapter of DSM-5 [24]. Previously denominated as dysmorphophobia, BDD is a severe psychiatric condition characterized by a recurring and persistent concern with an imagined or minor defect in physical appearance, focused on a specific body part. Common examples would be nose, hair, freckles, breast size, or the body shape as a whole [40]. The perceptions are intrusive, unwanted and usually hard to avoid or control [41, 42]. These include the “mirror gazing” which affects the large majority of those affected by BDD with reports of patients spending up to 11 hours per day looking at themselves in the mirror [43]. BDD is under recognized and often not diagnosed [42]. The negative impact of untreated BDD to the patient extends to most aspects of a person’s life and ultimately their mental health, as evidenced by the frequent associations with severe depression, suicidal ideation as well as both functional and social impairment [44, 45].

The vulnerability of individuals with tendencies towards EA and BDD, has been further challenged by the increasing prevalence of websites selling supplements as well as performance and image enhancing drugs (PIEDs) [46, 47]. Whilst anabolic-androgenic steroids (AAS) have been studied since the 1980s [48], an increasingly wide range of fitness enhancing products are advertised online with misleading marketing strategies, such as drastically improving one’s appearance in a faster and ‘safer’ way than traditional medicines or methods [49, 50, 51]. This market is poorly regulated [52, 53, 54] with an estimation that between 26% and 42% of these products have been contaminated with biologically-active ingredients. Consequently those purchasing these products are unware of the numerous health risks associated with their consumption [55, 56, 57, 58].

This study aims to explore the complex relationship among these different psychopathological aspects by measuring the level of EA in fitness settings and exploring the potential association with other psychopathological issues (appearance anxiety and self-esteem) and the use of fitness enhancing products in specific and potentially high-risk settings internationally (Italy, United Kingdom, Netherlands & Hungary). It is hypothesized that participants who report symptoms of EA report significant body-related anxiety as well as low self-esteem, and therefore represent the population with the highest chance of consuming fitness enhancing products to improve their fitness performance and consequent body image.

## Methods

### Recruitment

Participants were over 18 years old with membership to a fitness club. To recruit a suitable sample, a snowballing technique was used. An invitation to participate in the study was initially sent to selected fitness clubs that varied both in terms of the members they attracted (e.g. membership price) and the range of activities in their programs. In order to increase the validity of the sample fitness clubs were selected in different areas according to socio-economic criteria. Those with an interest in the study were redirected to the Keep Fit website (https://humanenhancementdrugs.com). Some participants completed questionnaires in front of a researcher inside the fitness club (35% in UK; 90% in Italy), whereas others participated via fitness/bodybuilding clubs mailing lists and their social media platforms (65% in UK; 10% in Italy; 100% in the Netherlands; 100% in Hungary). Data were collected from April 2015 to January 2016.

### Instruments

A questionnaire covering the following areas was designed: a) demographic information (age, gender, occupation, type of exercise/sport in the gym); b) measurement of the level of EA, self-esteem and appearance anxiety using the following psychometric scales: Exercise Addiction Inventory [59], Rosenberg Self-esteem Scale [60] and Appearance Anxiety Inventory [61]; c) supply and use of supplements to enhance fitness performance.

The Exercise Addiction Inventory [62, 63, 64] consists of six questions based on the six general components of addiction (salience, mood modification, tolerance, withdrawal symptoms, social conflict, and relapse). The responses are rated on a 5-point Likert scale ranging from 1 (*strongly disagree*) to 5 (*strongly agree*). A sum score is calculated (out of a maximum 30 points), where a score ≥ 24 indicates probable EA. With regard to the psychometric properties of the Exercise Addiction Inventory various studies have demonstrated the scale to be both valid and reliable [65, 66]. Furthermore, a recent cross-cultural re-evaluation of the Exercise Addiction Inventory in five countries showed that despite inter-country differences this instrument assesses EA with good reliability and validity [67].

The Appearance Anxiety Inventory [68, 69, 70] is a 10- item self-report questionnaire for measuring the frequency of avoidance behaviour and threat-monitoring (e.g. checking, self-focussed attention) that are characteristic of a response to a distorted body image; each item is scored from 0 (‘not at all’) to 4 (‘all the time’). The Appearance Anxiety Inventory was developed as a tool to measure change in psychotherapy patients suffering from BDD. Each item is scored on a five-point Likert scale ranging from 0 (not at all) to 4 (all the time), yielding one summary score with a maximum 40 with higher scores indicating a higher occurrence of appearance anxiety.

Rosenberg self-esteem scale [71, 72] intends to measure a single dominant factor representing global self-esteem. The Rosenberg self-esteem scale [9] has five positively and five negatively worded items. Its dimensionality was evaluated in a meta-analysis of 23-studies representing a total sample of 32,491 participants. The 2-factor structure with a positive self-esteem factor defined by five positively worded items and a negative self-esteem factor defined by five negatively worded items was generally supported [72].

Finally, the supply and use of fitness supplements was assessed by non-validated questions formulated by the researchers. In order to capture a wider spectrum of substances, the term “product” was used rather than, “supplement”, “enhancement drug” or “medicine”. Listed products were chosen after consultation with a network of experts, which included sport nutritionists, clinicians and sample representatives, who also supported the initial pilot of the questionnaire.

The survey questionnaire was made available online via Qualtrics [73, 74] and collected data were stored on a secure platform at the University of Hertfordshire.

### Statistical power and methods

With a sample size of 1711 and allowing for a 2% change in R^2^, 10 predictor variables and an alpha = 0.025 renders power greater than 9%. Data were processed with SPSS v23 and utilised descriptive analysis to: understand the sample [age, gender, body mass index, occupation, level of sport engagement]; use of products, source of products, motivation for using products, and their side-effects; Exercise Addiction Inventory, Appearance Anxiety Inventory and Rosenberg Self-Esteem scales. Chi-square was used to compare the data between male and female respondents, whilst ANOVA was used to evaluate possible gender differences in the different scales. A logistic regression was used to investigate the association between the use of products and a number of variables, including age (as a linear variable), Appearance Anxiety Inventory (considering a cut-off of 19, which detects a clinical presence of BDD [62, 63, 64]), Exercise Addiction Inventory (which suggests the presence of EA when score is above 24 points) [64, 67], RSE (as a linear variable); the analysis developed a model valid for the Keep Fit respondents, also distinguishing between male and female subjects.

### Ethics and privacy

The study was approved by the Human Sciences Ethics Committee at the University of Hertfordshire (HSK/SF/UH/00104). Each interviewee was asked to formally consent prior to participation. All responses were anonymous, securely stored and made accessible only to members of the research team.

## Results

Overall 1711 participants were recruited from the United Kingdom (377), Italy (494), Netherlands (189) and Hungary (651) (Table 1). The sample consisted of both female (66.3%) participants, and had a mean age of 30 (±10.26). Most respondents were employed (63.6%), while 29% were students, 5.3% unemployed and 1.2% retired at the time of the survey. The overall sample was engaged in a heterogonous class of fitness activities, including walking (53.3%), running (79.2%) and body weight exercises (28.7%). Overall sample characteristics are highlighted in Table 1.

**Table 1:** Sample description (N = 1711) with specification of demographics and types of physical activities.

General information (n = 1711)		n	Percentage	Male		Type of activities*	
Country	Hungary	651	38%	11.4%		Walking	53.3%
	Italy	494	28.9%	48%		Running	79.2%
	UK	377	22%	46.9%		Body-lifting	28.7%
	Netherlands	189	11%	47.1%		Lift- weights	27.2%
						Swimming	19.6%
Age	m = 30.17± 10.26					Hockey	17.9%
						Riding	16.9%
						Hiking	12.8%
Gender	Male		577	33.7%		Gymnastics	11.3%
	Female		1134	66.3%		Football	8.9%
						Yoga	8.7%
						Bike fast	8.6%
						Aerobics	8.6%
						Resistance	8.4%
Occupation	Employed		1091	64.3%		Martial arts	7.8%
	Student		496	29.2%		Skipping Rope	7.0%
	Unemployed		89	5.2%		Volleyball	6.7%
	Retired		20	1.2%		Rugby	5.7%
						Basketball	4.5%
						Tennis	4.3%

* Multiple choice

### Exercise Addiction, Appearance Anxiety and self-esteem

As shown in Table 2, an average score of 18.51±4.2 was reported in the Exercise Addiction Inventory with no significant differences between male and female, while EA was detected in 11.7% of the overall sample, considering the cut-off of the scale (24 points) as suggested in literature [62, 63]. The latter was higher amongst male (15%), who scored over 24 points in the Exercise Addiction Inventory.

**Table 2:** Exercise Addiction Inventory (EAI), Appearance Anxiety Inventory (AAI), Rosenberg Self-Esteem (RSE) scores and use of fitness supplements with specification of gender differences.

	Sample	Male	Female		Over Cut Off		
**EAI**	m = 18.51±4.2	m = 18.44±4.38	m = 18.65±3.97	n.s.	**11.7% (n = 191)**	Male = 81 (15%) Female = 110 (10%)	χ2 = 8.25 p<0.001
**AAI**	m = 18.14±5.7	m = 18.07±5.87	m = 18.28±5.58	n.s.	**38.5% (n = 577)**	Male = 108 (21.4%) Female = 468 (47.2%)	χ2 = 93.87 p<0.001
**RSE**	m = 12.33±2.48	m = 12.19±2.53	m = 12.58±2.37	f = 8.86 p<0.001			
**Fitness supplements**	Yes 39.8% (n = 657)	Yes 51.3%	Yes 34.2%	χ2 = 44.47 p<0.001	Note: Results seem influenced by country		Hungary Yes 52.3% (n = 332) χ2 = 155 p<0.001

Note: m = mean; χ2 = chi square; f = ANOVA

Despite a higher prevalence in males (15% vs 10% in females) scoring above the cut-off point of 24 [62, 63], no significant differences were found according to gender amongst the scores in the Exercise Addiction Inventory.

The Appearance Anxiety Inventory indicated an alarmingly high prevalence of appearance anxiety and low body satisfaction across the four countries. On the Appearance Anxiety Inventory, 38.5% of the overall was found at risks of BDD [62, 63] (Table 2). Low levels of self-esteem were also recorded on the Rosenberg Self-Esteem. As better highlighted in Table 3, the results were statistically different across the countries (p<0.001) with the Netherlands and Hungary respectively, showing the highest levels in the Exercise Addiction Inventory and Appearance Anxiety Inventory (20.9% and 51.5% over the cut-off).

**Table 3:** Exercise Addiction Inventory (EAI) and Appearance Anxiety Inventory (AAI) results per country.

	Netherlands	Hungary	UK	Italy	
EAI over cut-off (scored over 24)	20.9%	9.3%	16.1%	7.9%	X = 31.53 p<0.001
AAI over cut-off (scored over 19)	38.1%	51.5%	30.0%	29.5%	X = 64.29 p<0.001
					

A logistic regression model was used to predict the use of fitness products. The independent covariables used in this model were: age (linear variable); Rosenberg Self-Esteem scores (low self-esteem as a linear variable); EA (suggested by a score of 24 or above in the Exercise Addiction Inventory) and appearance anxiety (suggested by a score of 19 or above on the Appearance Anxiety Inventory, indicating a clinical risk for BDD). All the variables were tested in three models one for the entire sample and the other two according to gender. In all three models, the main predictor for the consumption of fitness products resulted to be EA. The EA odds ratio across the sample is 3.03; 95%; I.C 2.1–4.2; p<0.005. The same variable was the strongest predictor for the use of fitness products in both the other two models. The male’s EA odds ratio is 3.2; 95% I.C.1.8–5.8; p<0.005, while female’s odds ratio for EA is 2.5; 95%; I.C.1.6–3.8; p<0.005. In the female group other important covariable factors included appearance anxiety (Appearance Anxiety Inventory), odds ratio 1.59; 95% I.C. 1.2–2.1; p<0.001 and to a lesser extent self-esteem (Rosenberg Self-Esteem scale) with an odds ratio of 1.08; 95%; I.C. 1.025–1.152; p<0.005.

### Use and types of fitness supplements

Respondents who declared the use of fitness supplements scored significantly higher (p<0.001) in both the Appearance Anxiety Inventory and the Exercise Addiction Inventory (Table 4) with an average score of m = 19.62 in the Exercise Addiction Inventory, m = 18.91 in the Appearance Anxiety Inventory scale and m = 12.27 in the Rosenberg Self-Esteem scale. However, no significant difference was recorded in the Rosenberg Self-Esteem scale. Across the sample, a higher prevalence of EA was found among those using fitness enhancing products compared to non-users (m = 19.62±4.24 vs 17.78±4.24 p<0.001) across both the male and the female sample (Table 4).

**Table 4:** Fitness supplements users vs non-users: Differences considering to Exercise Addiction Inventory, Appearance Anxiety Inventory, Rosenberg Self-Esteem and their country.

	Users (n = 657)	Non Users	
EAI	m = 19.62±4.24	m = 17.78±4.24	f = 75.89; p<0.001
AAI	m = 18.91±5.88	m = 17.63±5.62	f = 17.99 p<0.001
RSE	m = 12.27±2.52	m = 12.38±2.44	n.s.
			
Hungary	52.3%	χ2 = 155 p<0.001	
Netherlands	52.2%		
UK	41.3%		
Italy	16.9%		

Note: m = mean; χ = chi square

The respondents specified the type of products used to enhance their fitness goals (Table 3), which included supplements (e.g. proteins 63.3%, vitamins 52.5%, amino acid 39.1%), caffeine (29.8%), teas (12.8%), mineral salts (13.1%), but also steroids (5.9%), diuretics (4.9%) or thyroid hormones (3%). 2.3% declared the use of amphetamine-like products and 1.1% had taken sibutramine, an appetite suppressant that has been withdrawn from most markets as it increases risk of cardiovascular events and strokes. The sample purchased these products in fitness shop (48.6%) and online (31.3%), which were also the main sources of information on these products. Interestingly participants declared the presence of side-effects (8.6%), but only a small percentage referred to a medical doctor (4.5%) (Table 5).

**Table 5:** Use of fitness supplements: Type, source of purchase and source of information.

Type of Fitness Product * (n = 657)	Percentage		Source of purchase	
Proteins	63.3%		Fitness Shop	48.6%
Vitamins	52.5%		Online	31.3%
Aminoacids	39.1%		Pharmacy	8.3%
Caffeine products	29.8%		Food Store	3.8%
Fish Oil	29.7%		Other source	8.0%
Mineral salts	13.1%			
Teas	12.8%			
Herbal products	9.4%		**Source of information**	
Antioxidants	8.2%		Online	41.4%
NO	8.2%		Friends	18.9%
Ginseng	7.9%		Personal Trainer	14.6%
Guaranà	7.0%		Magazine	10.5
Steroids	5.9%		Medical professionals	4.5%
Diuretics	4.9%		Family	3.7%
Thyroid hormones	3%		Other	6.3%
Laxatives	2.3%			
Amphetamine-like products	2.3%			
GH	1.8%		**Experienced side-effects**	
Sibutramine	1.1%		Yes	8.6%

* Multiple choice

### Logistic regression models using Exercise Addiction Inventory scores to predict the use of fitness supplements

Three different logistic models were created according to the gender of participants (Table 6).

1. Across the whole sample, EA emerged as a strong predictor of the consumption of fitness supplements. This risk appeared to be over three times higher in those reporting Exercise Addiction Inventory scores over the cut-off (p<0.001, OR = 3.03 with a Confidence Interval (CI) ranging from 2.15 to 4.28.
2. The risk of using supplements was much higher in the male sample reporting Exercise Addiction Inventory score over the clinical cut-off (OR = 3.25, CI 1.81–5.86). In those cases where EA was identified, the risk of using sport supplements was over five times higher, making EA the strongest predictor for this group.
3. Although EA was also a significant predictor for supplement use, but to a lesser extent in the female group (OR = 2.50), two additional psychopathological factors emerged: Appearance Anxiety (OR = 1.5; CI 1.20–2.12) and low self-esteem as measured by the Rosenberg Self-Esteem scale (OR = 1.08; CI 1.02–1.15).

**Table 6:** Logistic regression models with specification of gender.

**Model I: fitness supplements** **(whole sample)**		B	ES	Wald	df	Sig	Odds Ratio (OR)	Confidence Interval (CI)	
								Min	Max
	Age	.000	.006	.000	1	.989	1.000	.989	1.011
	RSE total	.030	.023	1.796	1	.180	1.031	.986	1.078
	**EA over the Cut Off (1)**	**1.111**	**.175**	**40.061**	**1**	**.000**	**3.037**	**2.153**	**4.283**
	AAI over the Cut off (1)	.243	.116	4.352	1	.037	1.275	1.015	1.602
	Constant	1.118	.362	9.526	1	.002	.327		
									
**Model II: fitness supplements** **(male)**	Age	.010	.009	1.167	1	.280	1.010	.992	1.027
	RSE total	-.073	.037	3.810	1	.051	.930	.864	1.000
	**EA over the Cut Off (1)**	**1.182**	**.300**	**15.508**	**1**	**.000**	**3.259**	**1.810**	**5.868**
	AAI over the Cut off (1)	.455	.240	3.592	1	.058	1.577	.985	2.525
	Constant	-.852	.608	1.963	1	.161	.427		
									
**Model III: fitness supplements (female)**									
	Age	-.006	.008	.596	1	.440	.994	.980	1.009
	RSE total	.083	.030	7.827	1	.005	1.087	1.025	1.152
	**EA over the Cut Off (1)**	**.918**	**.226**	**16.541**	**1**	**.000**	**2.504**	**1.609**	**3.898**
	**AAI over the Cut off (1)**	**.469**	**.145**	**10.389**	**1**	**.001**	**1.598**	**1.202**	**2.125**
	Constant	1.297	.466	7.740	1	.005	.273		
									

### Country and gender differences

Considering the cross-cultural nature of our study, interesting differences emerged from the comparison among the participating countries with relevant gender differences:

- Exercise Addiction Inventory. The presence of EA was highest in the Netherlands (20.9%), followed by the United Kingdom (16.1%), Hungary (9.3%) and Italy (7.9%) (Table 3). Overall, EA was found among 15% of male respondents and 10% of female respondents, who scored higher than 24 in the Exercise Addiction Inventory (Table 2).
- Appearance Anxiety Inventory. The risks of BDD was highest in Hungary (51.5%), Netherlands (38.1%), the UK (30%) and Italy (29.5%) (Table 3). Reported scores were higher among the female (47.2%) than the male (21.4%) sample (Table 2).
- Use of products. Respondents in Hungary (52.3%) and Netherlands (52.2%) declared a higher use of fitness enhancing products, followed by the UK (41.3%) and Italy 16.9% (Table 4). The consumption was mainly reported among the male sample (51.3%) (Table 2).

## Discussion

In Western cultures dominated by physical appearance, bodies are increasingly objectified and perceived as a machine that can be modified and manipulated at will. Such a mechanical conception of the human body and its functions fits a dualistic way of thinking, where there is little or no significant interaction between mind and body [75, 76]. Caught by frantic lifestyles, daily routines, rapid transportation and communication, individuals feel increasingly disconnected from the environment around them, chasing an “ideal” body [76]. This is implicit in the claim that everybody has the power to choose their appearance, which is constantly advertised by social and other media platforms [77]. Such phenomenon has been increasingly linked with appearance anxiety and other psychopathological features, resulting in the need to explore this trend in gyms across Europe. As a result, this study revealed the presence of EA as well as a previously unexplored association with appearance anxiety, low self-esteem and the use of a variety of fitness supplements taken without medical consultation.

Although the concept of EA is still not well defined in the literature, and we still do not have robust evidence as to its incidence nor prevalence, some initial studies highlighted its occurrence among sporting sub-groups, such runners (26%) and triathletes (52%) [78], and rarely among other sport disciplines [79]. Owing the current terminological confusion, the small-scale investigations, and the heterogeneity of the instruments used in the assessment of EA, the emerging figures for prevalence across these studies differ widely [80, 81]. However, despite such variance, the reported levels of EA, confirm levels of EA far above the average observed across the general population (0.3–0.5%) [82].

In our study a medium level of EA was recorded across the whole sample (Table 2) indicating higher exposure to physical injuries and withdrawal symptoms, like depression, anxiety and mood swings. Higher levels of EA of clinical concern (over the clinical cut-off score) were found among the 11.7% of the sample, especially male (p<0.001) [62, 63].

Overall participants were concerned about their physical appearance. This was confirmed by the high risk of manifesting a BDD among the sample that was significantly higher amongst females (Table 2). This result was much higher not only than the scores reported among the general population (where BBD prevalence ranges from 0.7% to 2.3%), but also of those found among at risk populations, such as individuals undergoing aesthetic surgery, specific dermatology and rhinoplasty patients (6–15%) [83]. As far as we know, this is the first evidence highlighting such an alarming presence of population at risk of BDD in fitness settings, especially among the female sample. This result might be influenced by the fact that the engagement with exercise is often considered appearance-related [84] and that exercise settings, such as fitness centres, can “induce self-objectification by creating an environment that promotes a focus on outward appearance and unrealistic physical body ideals” [85]. People who actively work out will therefore generally score higher on being dissatisfied with their body when compared with others in society. Overall, we observed a high rate of participants who engaged in professional and amateur bodybuilding, particularly in the Netherlands, which could explain the higher rate of body dissatisfaction within this group. It would of interest in future investigations to include an assessment of other image-related disturbances, such as muscle dysmorphia, a psychiatric disorder characterized by the preoccupation with the idea that one's body is not lean and muscular enough that remains undereported.

A large percentage (39.8%) of respondents reported the use of different fitness products (Table 2). Although both the positive and negative aspects of the use of supplements, such as proteins, caffeine, creatine and steroids have been documented in the literature [86, 87], still more work needs to be carried out on the unwanted side-effects, which were reported by 8.6% of our sample. In our sample, these mainly included cases of irritation, tachycardia, insomnia, mood changes, diarrhoea, libido changes. The Italian sample showed the lowest percentage of EA (7.9%) and fitness products’ consumption. Another study in the country [88], found similar level of EA (8.5%) among a student sample. It could be argued that a Mediterranean diet [89, 90, 91], which has been associated with better health outcomes [92], especially in terms of weight loss and glycaemic control [93], might have impacted the lower level of EA and the consumption of fitness products.

The diffusion of fitness products, especially PIEDs, remains marginally studied and regulated [52, 53, 54], and deserves prompt responses from authorities informed by evidence base [49, 94, 50]. Online advertising (41.4%) and peer-pressure (14.6%) appeared to be key drivers for the use of sporting and muscular enhancing products at gyms. Medical supervision for the use of these substances was sought only by 4.5% of the sample. Although consultation with a doctor is not strictly required for some dietary supplements, this is mandatory for other products such as steroids, hormones, diuretics, and their use must be clinically supervised. In addition, often captivated by misleading marketing strategies, users might be unaware of the risk of contamination of their purchases with psychoactive and potential highly toxic undisclosed ingredients [55, 56, 57]. As observed in other studies [95, 96, 97], the use of fitness supplements within gym culture is often perceived to be safe, acceptable and necessary in the pursuit of body/fitness ideals, while disregarding any potentially associated harms. Mistrust of medical professionals and stigma might also have a role in facilitating such risk-taking behaviours [98, 99, 100]. Such a hazardous intake, which might lead to damaging physical and psychiatric consequences, has also been observed among individuals using sexual and weight loss enhancers. This applies especially to those advertised online or elsewhere as having “natural” ingredients as these were perceived as “safer” than prescription drugs and therefore taken without medical supervision [94, 101].

As an important contribution to the field, the applied logistic regression models revealed for the first time an association between the consumption of fitness supplements and the level of EA across sample, suggesting a predictability of use. Although preliminary, these findings indicate that intake of fitness enhancing products could be motivated by such underlying psychopathological and image-disturbance features. The statistical significance of EA was particularly high for males, where the risk of fitness supplements intake was from 1.8 to 5.8 times higher than that reported among females (Table 6). The desire to achieve a ‘V shaped’ muscular body might make vulnerable individuals not only more inclined to use supplements, including illicit drugs, but also to suffer from Muscle Dysmorphia. Conversely, the substance intake by the female group was more influenced by appearance anxiety and the self-esteem. Such gender differences underlying the intake of fitness supplements must be explored very carefully in future analysis.

Results from our work may have been affected by unreported psychiatric or medical disorders. It is well known for instance that excessive exercise can be a symptom of anorexia nervosa, as a mechanism that promotes and sustains the core pathologic drive for thinness [102]. Our multidimensional evaluation and preliminary considerations align with current dimensional and trans-diagnostic approach to psychiatric disorders and dysfunctional behaviours, hypothesizing that different disorders might share some clinical and neurochemical characteristics as well as biological and personal vulnerabilities [103]. Investigating unexplored dimensions such as impulsivity, compulsivity and perception of the self, both in general and clinical populations, might open new directions for future research aimed at developing new knowledge and effective treatment for related conditions.

Overall our findings need to be considered within a context of aesthetic idealisation, rather than a search for wellbeing, or a hedonistic pursuit of pleasure. Fitness goals are strongly influenced by the perception of one’s reflection in the mirror, or the number of “likes” for one’s picture on social networking. This justifies the urgency for more targeted prevention strategies and policy responses addressed to this newly identified at risk-population as well as the need to inform clinicians and professionals working in both the sport and the aesthetics areas, who could identify potential patients requiring referral to specialized treatment.

As we have seen, the study of EA as an emerging phenomenon in society remains contradictory. The lack of consistency in the limited evidence base has been attributed to a number of factors, including variations in the study design and methods, use of non-validated scales, the self-reported nature of various study focusing on specific sports or physical activities. We also remain at the early stages of measuring the prevalence and the incidence of EA in society as well as of considering the complex and interrelated motivations and drivers for such compulsive behaviour in the gym culture and beyond.

Although Keep Fit made a significant contribution to the field, authors remain aware of the study limitations, which can be summarised as follows: (a) the assessment of a non-stratified population with different recruitment procedures among countries; (b) the absence of a structured psychiatric interview able to assess the emerged underlined pathologies; (c) the use of fitness products was self‐reported and not validated by any biological testing; (d) the lack of information on the frequency and duration of the exercise and use of fitness products.
